# Grape Seed Procyanidins and Cholestyramine Differentially Alter Bile Acid and Cholesterol Homeostatic Gene Expression in Mouse Intestine and Liver

**DOI:** 10.1371/journal.pone.0154305

**Published:** 2016-04-25

**Authors:** Rebecca M. Heidker, Gianella C. Caiozzi, Marie-Louise Ricketts

**Affiliations:** Department of Agriculture, Nutrition and Veterinary Sciences, University of Nevada Reno, Reno, Nevada, United States of America; University of Navarra School of Medicine and Center for Applied Medical Research (CIMA), SPAIN

## Abstract

Bile acid (BA) sequestrants, lipid-lowering agents, may be prescribed as a monotherapy or combination therapy to reduce the risk of coronary artery disease. Over 33% of adults in the United States use complementary and alternative medicine strategies, and we recently reported that grape seed procyanidin extract (GSPE) reduces enterohepatic BA recirculation as a means to reduce serum triglyceride (TG) levels. The current study was therefore designed to assess the effects on BA, cholesterol and TG homeostatic gene expression following co-administration with GSPE and the BA sequestrant, cholestyramine (CHY). Eight-week old male C57BL/6 mice were treated for 4 weeks with either a control or 2% CHY-supplemented diet, after which, they were administered vehicle or GSPE for 14 hours. Liver and intestines were harvested and gene expression was analyzed. BA, cholesterol, non-esterified fatty acid and TG levels were also analyzed in serum and feces. Results reveal that GSPE treatment alone, and co-administration with CHY, regulates BA, cholesterol and TG metabolism differently than CHY administration alone. Notably, GSPE decreased intestinal apical sodium-dependent bile acid transporter (*Asbt*) gene expression, while CHY significantly induced expression. Administration with GSPE or CHY robustly induced hepatic BA biosynthetic gene expression, especially cholesterol 7α-hydroxylase (*Cyp7a1*), compared to control, while co-administration further enhanced expression. Treatment with CHY induced both intestinal and hepatic cholesterologenic gene expression, while co-administration with GSPE attenuated the CHY-induced increase in the liver but not intestine. CHY also induced hepatic lipogenic gene expression, which was attenuated by co-administration with GSPE. Consequently, a 25% decrease in serum TG levels was observed in the CHY+GSPE group, compared to the CHY group. Collectively, this study presents *novel* evidence demonstrating that GSPE provides additive and complementary efficacy as a lipid-lowering combination therapy in conjunction with CHY by attenuating hepatic cholesterol synthesis, enhancing BA biosynthesis and decreasing lipogenesis, which warrants further investigation.

## Introduction

Currently one in every four deaths in the US is attributable to cardiovascular disease (CVD) [1]. Regulation of two controllable CVD-associated risk factors, namely serum cholesterol and triglyceride levels, is tightly linked to BA homeostasis. BAs, in addition to their established role in digestion, function as signaling molecules with systemic endocrine effects. BAs regulate not only their own uptake and synthesis, but also cholesterol and triglyceride homeostasis [2–4]. Consequently, modifications in BA-activated signaling pathways have become an attractive therapeutic target for treating hypercholesterolemia and hypertriglyceridemia. Identifying potential gene regulatory interactions between pharmaceutical interventions and natural treatments used in the amelioration of risk factors associated with CVD is important.

BAs are synthesized from cholesterol in the liver, secreted into bile, stored in the gall bladder, and post-prandially released to facilitate dietary lipid and fat-soluble vitamin absorption. They are reabsorbed in the terminal ileum and returned to the liver via the portal vein, in a process called enterohepatic recirculation [5]. Reuptake of BAs is facilitated via the apical sodium-dependent bile acid transporter (Asbt) [6], the expression of which is inversely regulated via BA activation of the farnesoid x receptor (Fxr) [7]. BAs are then transported to the basolateral membrane by ileal bile acid binding protein (Ibabp) [8] and released into portal circulation through the organic solute transporters α/β (Ostα/β) [9]. Typically 95% of the BAs are returned to the liver and eventually released back into the gall bladder, with the remaining 5% being replenished via endogenous biosynthesis from cholesterol [6].

The *Cyp7a1* gene, encoding cholesterol 7α-hydroxylase (the rate limiting enzyme in the classical (or neutral) pathway for BA biosynthesis [10]) is regulated via the gut-liver axis by intestinally-derived fibroblast growth factor 15 (*Fgf15*) [11]. *Fgf15* is induced via BA activation of Fxr, secreted into portal circulation, and upon reaching the liver, binds to Fgf receptor 4 (Fgfr4), signaling through c-Jun N-terminal kinase (Jnk) to repress *Cyp7a1* expression [12]. When BA levels are depleted, Fgf15 expression is decreased and Cyp7a1 is increased to initiate BA synthesis [11, 13]. *Cyp8b1*, encoding sterol 12α-hydroxylase, introduces a hydroxyl group at position 12 of the steroid nucleus, leading to the generation of cholic acid (CA) [14, 15]. Cyp7a1 governs the BA pool size, whereas Cyp8b1 is crucial for determining the BA pool composition [16, 17]. The classical pathway for BA synthesis accounts for at least 75% of the total BA pool [18]. Sterol 27-hydroxylase, encoded by the *Cyp27a1* gene, is important for the production of both CA and chenodeoxycholic acid (CDCA) [19]. In the alternative (or acidic) pathway, oxysterols generated by sterol 27-hydroxylase are hydroxylated at the 7α position by oxysterol 7α-hydroxylase (Cyp7b1), before eventually being converted to CDCA [20]. Increased conversion of cholesterol into BAs ultimately leads to a decrease in intracellular cholesterol stores [21]. This results in increased low density lipoprotein (LDL) receptor (*Ldlr*) expression, leading to increased LDL uptake and decreased plasma LDL levels [21, 22].

To maintain homeostasis, the body must replenish intracellular cholesterol pools via increased cholesterol synthesis, which occurs largely in the liver and intestine [23, 24]. Synthesis of cholesterol is controlled by the transcription factor sterol regulatory element binding protein 2 (encoded by the *Srebf2* gene), which positively regulates cholesterol synthesis via 3-hydroxy-3-methylglutaryl-CoA synthase 1 (Hmgcs1) and 3-hydroxy-3-methylglutaryl-CoA reductase (Hmgcr) [25]. Newly synthesized cholesterol is esterified by acetyl-CoA acetyltransferase 2 (Acat2) [26], and loaded onto apolipoprotein (apo)-B containing lipoproteins (chylomicrons in the intestine and VLDL or LDL in the liver) via microsomal triglyceride transfer protein (Mttp) [27–29]. Scavenger receptor class b, member 1 (Scarb1) is important for dietary cholesterol uptake and has been implicated in increased chylomicron synthesis in the intestine [30]. Additionally, intestinal ATP-binding cassette, sub-family a, member 1 (Abca1) mediates the transfer of cholesterol and phospholipids to apolipoprotein A1 and ApoE, facilitating the formation of nascent HDL [31].

BAs also regulate TG homeostasis via Fxr activation, leading to increased expression of small heterodimer partner (Shp), which ultimately represses sterol regulatory element binding protein 1c (Srebp1c, encoded by the *Srebf1c* gene) [4]. Diminished expression of Srebp1c leads to repressed lipogenic gene expression, including fatty acid synthase (*Fasn*), acetyl CoA carboxylase 1 (*Acc1*), and stearoyl CoA desaturase (*Scd1*). In addition, Fgf15/19 (Fgf15 in mouse and its human ortholog FGF19) signaling leads to indirect suppression of hepatic Srebp1c activity, by increasing signal transducer and activator of transcription 3 (STAT3) phosphorylation and down-regulating peroxisome proliferator-activated receptor coactivator 1-beta (Pgc-1β) expression, thereby inhibiting Srebp1c transcriptional activity at the *Fasn* and *Acc* promoters [32, 33]. Also, Fgf15/19 signaling increases the atypical protein kinase isoform Czeta, PKCζ, leading to increased phosphorylation of Shp on Thr-55, which subsequently represses Srebp1c-mediated *Fas*n and *Acc* transcription [32, 34].

BA sequestrants have been used for over 40 years as a means to impact lipoprotein metabolism and lower serum cholesterol levels [21]. These agents bind BAs in the intestine and reduce transhepatic BA flux, leading to the accelerated conversion of cholesterol into BAs [35]. The consequential reduction in intracellular cholesterol stores initiates the activation of HMGCoA reductase, leading to increased *de novo* cholesterol synthesis. BA sequestrants may be prescribed to patients as a monotherapy or combination therapy with statins or other lipid-lowering agents to provide a more aggressive LDL-lowering regimen [36, 37]. CHY therapy may modestly increase TG levels, however, concentrations do not generally exceed the upper limit for the normal range [21]. Dramatic increases in TG levels usually occur during BA sequestrant therapy in individuals with a metabolic defect affecting the catabolism of TG-containing lipoproteins [21], and are not indicated for those patients with pre-existing hypertriglyceridemia [38].

We previously showed that GSPE functions in an Fxr-dependent manner [39], reduces enterohepatic BA recirculation, resulting in decreased serum cholesterol and triglyceride levels [40], and attenuates fructose-induced hypertriglyceridemia [41]. Currently over 33% of adults in the US utilize complementary and alternative medicine strategies [42], therefore it is possible that patients may take a grape seed extract in combination with a BA sequestrant, such as CHY. Consequently, this study was designed to gain further insight into the molecular regulatory effects of GSPE and CHY on BA, cholesterol and TG homeostatic gene expression when administered alone and in combination.

## Materials and Methods

All chemicals were obtained from ThermoFisher Scientific (Picastaway, NJ) unless otherwise stated. Grape Seed Procyanidin Extract (GSPE) was obtained from *Les Dérives Résiniques et Terpéniques* (Dax, France), and is comprised of procyanidin monomers (68.68 ± 0.02%), dimers (26.16 ± 0.01%) and trimers (5.16 ± 0.02%) [41].

### Animal care, diets and treatments

Mice were housed under standard conditions and all experimental procedures were approved by the local Institutional Committee for Care and Use of Laboratory Animals (IACUC) at the University of Nevada, Reno (Protocol# 00502). Age-matched groups of male C57BL/6 mice were used in all experiments, and were housed in the Laboratory of Animal Medicine (LAM) at the University of Nevada, Reno and provided access to chow and water *ad libitum*. Mice were purchased from Charles River Laboratories (Wilmington, MA) at 7 weeks of age and allowed to acclimate in the LAM for one week. At 8-weeks of age the mice were given either a control (standard chow, Harlan Teklad rodent diet 2019) or a 2% cholestyramine-supplemented diet (Harlan Teklad diet: TD.110785) for 4 weeks (n = 18 per group). Body weight for each mouse was recorded weekly. After 4 weeks, the mice in each group were randomly assigned to one of two treatment groups and orally gavaged with either vehicle (water) or GSPE (250 mg/kg) and terminated 14 hours later (n = 9 per experimental group). The four treatment groups were as follows: 1. **CON**: Control diet for 4 weeks followed by oral gavage with vehicle (water) for 14 hrs; 2. **GSPE**: Control diet for 4 weeks followed by oral gavage with 250 mg/kg GSPE for 14 hrs; 3. **CHY**: 2% cholestyramine-supplemented diet for 4 weeks followed by oral gavage with vehicle for 14 hrs; and 4. **CHY+GSPE**: 2% cholestyramine-supplemented diet for 4 weeks followed by oral gavage with 250 mg/kg GSPE for 14 hrs. The dose of procyanidins used is one-fifth of the no-observed-adverse-effect level (NOAEL) described for GSPE in male rats [43] and we previously showed that this dose reduces serum TG levels in normolipidemic C57BL/6 mice [39, 40, 44] and fructose-induced hypertriglyceridemic rats [41]. Blood was collected from the orbital plexus under isoflurane anesthesia, and intestines and livers were snap-frozen in liquid nitrogen and stored at –80°C until use. At the start of the 14 hr experiment mice were placed into clean cages, and feces were manually collected at the end of the study, air-dried and weighed.

### RNA isolation and gene expression analysis

Total RNA was extracted from tissues using TRIzol (Life Technologies) according to the manufacturer’s protocol. Complementary DNA (cDNA) was reverse transcribed using superscript III reverse transcriptase (Life Technologies), and real-time quantitative polymerase chain reaction (qPCR) was used to determine gene expression changes. qPCR was performed using a CFX96 Real-Time System (BioRad). Forward and reverse primers and probes were designed using Oligo Architect Software (Sigma-Aldrich) and obtained from Sigma-Aldrich or Integrated DNA Technologies. Primer and probe sequences can be found in S1 Table. Expression of *cyclophilin*, glyceraldehyde-3-phosphate dehydrogenase (G*apdh)* and TATA-binding protein (*Tbp)* and were used as endogenous controls. Target gene expression was normalized to the average of two or three endogenous control genes (intestine: *Gapdh* and *Tb*p; liver: *cyclophillin*, *Gapdh* and *Tbp*), and the ΔΔCt method was used to calculate the fold change in gene expression. Each sample was analyzed in duplicate.

### Plasma biochemical analyses

Serum triglyceride and total cholesterol levels were measured enzymatically using Infinity^™^ kits (ThermoFisher) according to the manufacturers’ instructions using 1.5 μl serum and 150 μL of reagent. Serum bile acid concentrations (20 μL per sample) were measured enzymatically using the Total Bile Acids Assay kit from Diazyme Laboratories. Alanine aminotransferase (ALT: (SGPT) Reagent Set, Cat. No.: A526-120) and aspartate aminotransferase (AST: (SGOT) Reagent Set, Cat. No.: A561-120) were measured using colorimetric based kits from Teco Diagnostic, according to the manufacturers’ instructions, using 100 μL of sample and 500 μL of reagent. All analyses were performed in triplicate using a Biotek Synergy HT microplate reader.

### Measurement of fecal bile acid, cholesterol, non-esterified fatty acid and total lipid excretion

To determine fecal bile acid excretion, a modified version of the method reported by Modica and colleagues [45] was used to measure the bile acid content, as previously described [40, 41]. Fecal cholesterol and non-esterified fatty acids were extracted as previously described [41], and cholesterol levels were measured using a colorimetric Infinity^™^ cholesterol assay kit and non-esterified fatty acids were quantified using a Wako diagnostics HR Series NEFA-HR (2) assay. Total fecal lipids were assessed as previously described [41] and results are expressed as mg lipid/g dry fecal weight.

### Statistical Analysis

One-way analysis of variance (ANOVA) with Holm-Sidak post-hoc analysis was employed to detect significant differences between groups. Treatment differences were considered statistically significant at p<0.05. All statistical analyses were performed using GraphPad Prism version 6.05 for Windows, GraphPad Software (San Diego, CA).

## Results and Discussion

Each individual mouse was weighed weekly during the 4 week dietary intervention period, and as shown in Table 1, the mice fed the 2% CHY-supplemented diet showed no significant differences in body weight compared to the control group at any time during the study.

**Table 1:** Average weekly mouse weight (g) during dietary intervention.

Week #	0	1	2	3	4
Control diet	23.7 ± 0.2	24.4 ± 0.3	25.2 ± 0.3	25.8 ± 0.3	26.5 ± 0.4
2% Cholestyramine	23.4 ± 0.3	24.5 ± 0.3	25.1 ± 0.3	25.5 ± 0.4	26.4 ± 0.4

Data represent mean ± SEM, n = 18 per diet.

### GSPE and cholestyramine differentially modulate bile acid uptake and transport

Our first aim was to determine the effects of GSPE, CHY and co-administration with CHY+GSPE on intestinal BA uptake and transporter gene expression in order to elucidate any differences and/or interactions at the transcriptional level.

**Fig 1:**
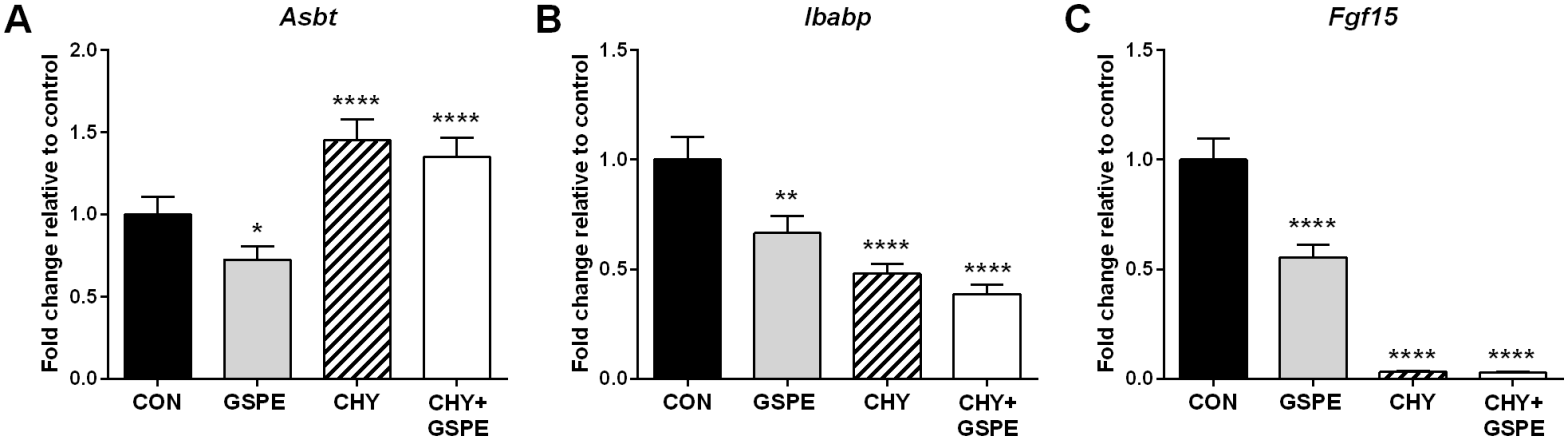
GSPE and cholestyramine differentially alter intestinal bile acid homeostatic gene expression. Gene expression changes were analyzed for (A) *Asbt*, (B) *Ibabp*, and (C) *Fgf15*. Statistical differences are shown as: *p≤0.05, ** p≤0.01, **** p≤0.0001.

As shown in Fig 1A, apical intestinal BA transport is regulated differently by GSPE and CHY. In agreement with our previous report [40], *Asbt* expression was significantly reduced by GSPE treatment, indicating decreased BA transport into the intestine. In contrast, CHY treatment robustly induced *Asbt* expression. Decreased apical BA uptake in both the CHY and CHY+GSPE groups, resulting from the BA binding action of this resin, is the probable explanation for the observed increase in *Asbt* expression. GSPE administration also caused a decrease in both *Ibabp* (Fig 1B) and *Fgf15* expression (Fig 1C), consistent with previous reports [40], while CHY and CHY+GSPE further reduced the levels of both of these genes. Reduced *Fgf15* expression following CHY treatment is also consistent with previous reports in human subjects who displayed an 87% reduction in serum FGF19 levels; and in rats where a >95% reduction in Fgf15 expression was observed [46].

### Cholestyramine and GSPE collectively increase hepatic bile acid biosynthesis

**Fig 2:**
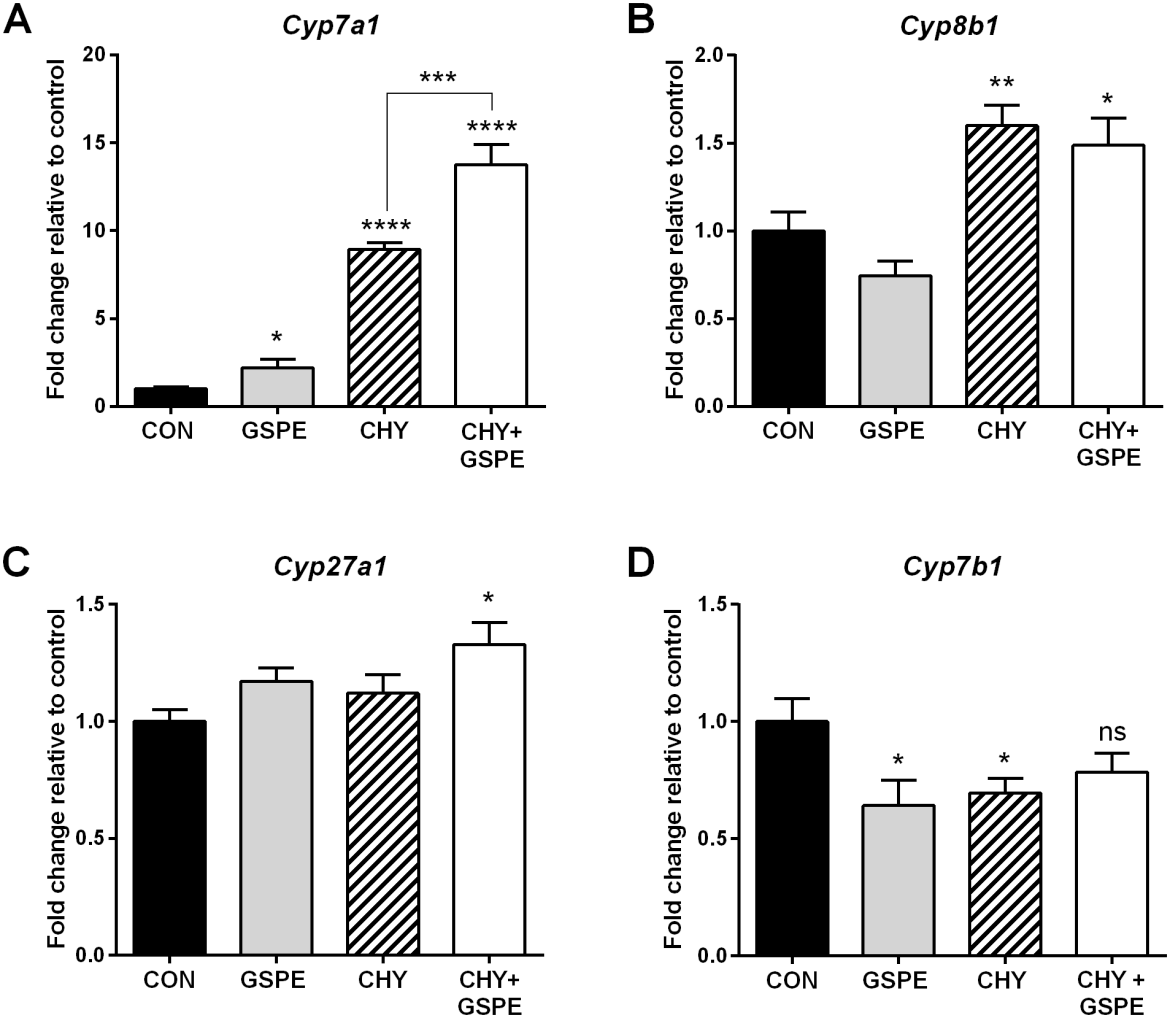
GSPE and cholestyramine induce the hepatic expression of genes regulating bile acid synthesis. Gene expression was analyzed for (A) *Cyp7a1*, (B) *Cyp8b1*, (C) *Cyp27a1*, and (D) *Cyp7b1*. Statistical differences are shown as: *p≤0.05, ** p≤0.01, ***p≤0.001, **** p≤0.0001.

Based on the finding that BA uptake into the intestine is differentially altered by CHY and GSPE through modulation of *Asbt* expression, we next examined their effect on hepatic BA biosynthetic gene expression. GSPE treatment upregulated *Cyp7a1* expression (Fig 2A) while CHY induced expression 8-fold compared to the control, facilitating increased BA biosynthesis to replenish those lost via the feces following administration. Interestingly, the CHY+GSPE group displayed a nearly 13-fold increase in *Cyp7a1* expression, compared to control, suggesting that GSPE, in combination with CHY, exerts an additive effect on *Cyp7a1* regulation. This may be linked to the reduced *Asbt* expression induced by GSPE in the intestine. Previous studies showed that *Cyp8b1* expression increases concomitant with *Cyp7a1* expression [47]. In agreement, *Cyp8b1* expression was increased by CHY and CHY+GSPE treatment. However, *Cyp8b1* expression was not significantly affected by GSPE treatment alone in this study (Fig 2B). *Cyp27a1* expression was not significantly altered by treatment with either GSPE or CHY alone, but was increased by CHY+GSPE, compared to control (Fig 2C). *Cyp7b1* expression was decreased following CHY or GSPE treatment, but no change was observed in the CHY+GSPE group, compared to control (Fig 2D). Overall the data indicates that BA biosynthesis is induced by all treatments compared to control.

### Intestinal cholesterol transport and synthesis are differentially regulated by GSPE and cholestyramine

**Fig 3:**
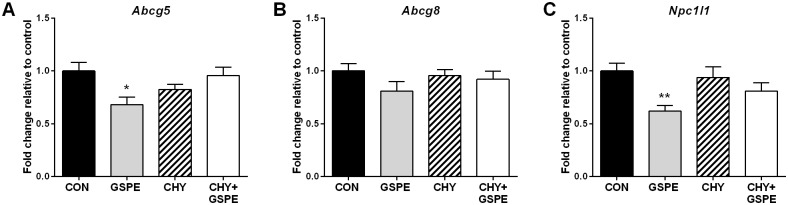
GSPE decreases the expression of intestinal apical cholesterol transporters, but not in combination with cholestyramine. Gene expression was analyzed (A) *Abcg5*, (B) *Abcg8*, and (C) *Npc1l1*. Statistical differences are shown as: *p≤0.05, ** p≤0.01.

BA and cholesterol homeostasis are tightly regulated along the gut-liver axis and based on the observed differences in intestinal BA uptake and hepatic BA biosynthesis, we next examined the effect on intestinal apical cholesterol transport and synthesis. GSPE administration significantly reduced *Abcg5* expression (Fig 3A), while *Abcg8* expression was not significantly changed (Fig 3B). Reduced Abcg5 expression following GSPE treatment may lead to reduced transport of intracellular cholesterol into the lumen of the intestine.

GSPE significantly decreased the expression of *Npc1l1* (Fig 3C), a crucial transporter for dietary cholesterol uptake, whereas CHY and CHY+GSPE had no such effect. These contrasting effects suggest that the cholesterol flux into the enterocyte may not be the regulatory step to control cholesterol absorption in the presence of CHY, in agreement with previous reports [48]. Curcumin, another dietary polyphenol, was also reported to decrease *Npc1l1* expression [49]. Therefore, it is possible that GSPE, by inhibiting *Npc1l1* expression, functions in a similar manner to inhibit intestinal cholesterol uptake, however, the exact mechanism by which this occurs warrants further investigation.

**Fig 4:**
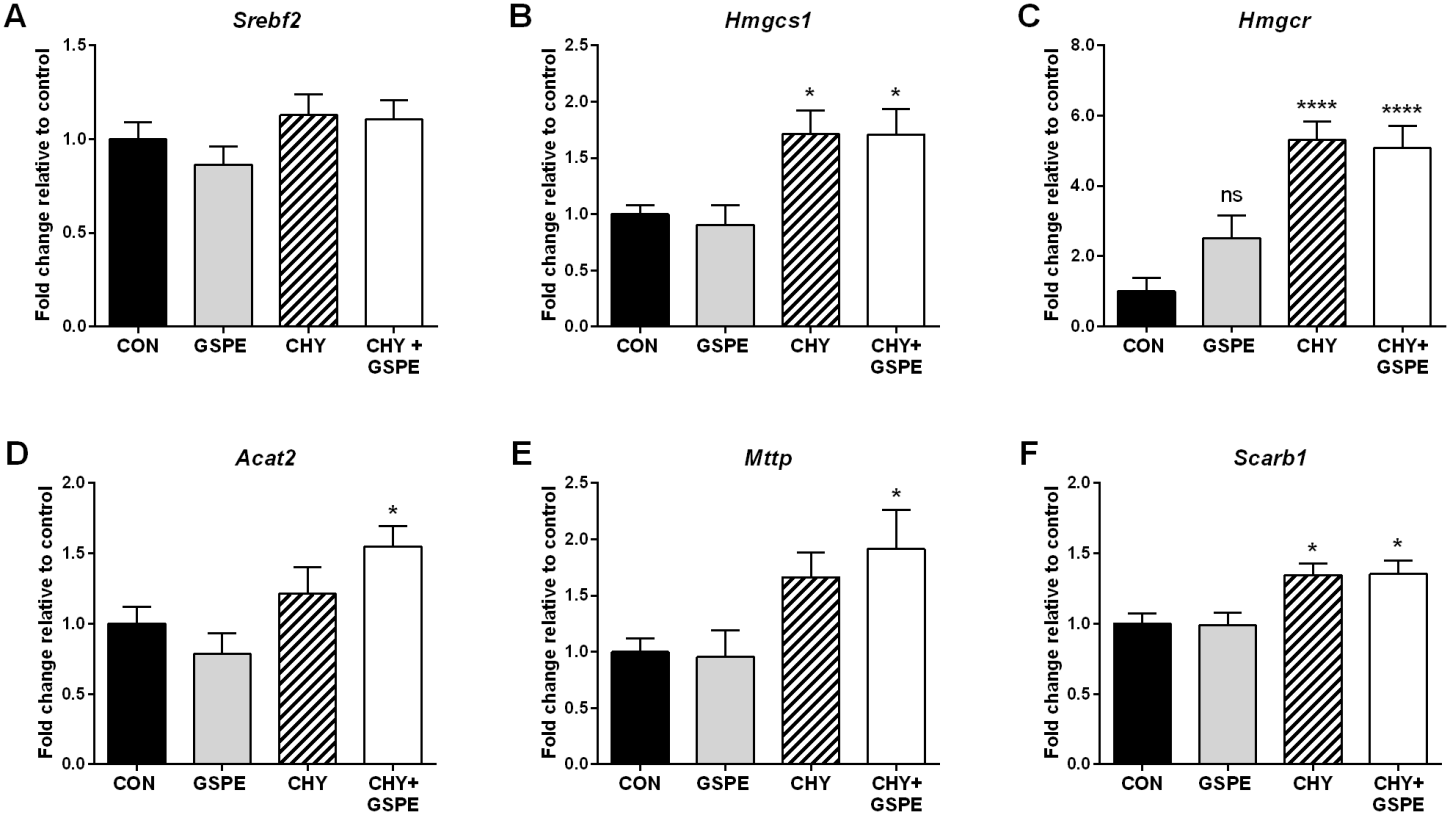
Effects on intestinal cholesterol synthesis and transporter gene expression following treatments. Gene expression was analyzed for (A) *Srebf2*, (B) *Hmgcs1*, (C) *Hmgcr*, (D) *Acat2*, (E) *Mttp*, and (F) *Scarb1*. Statistical differences are shown as: *p≤0.05, **** p≤0.0001.

As shown in Fig 4A, *Srebf2* expression was unchanged by any of the treatments, whereas CHY significantly increased the expression of genes responsible for intestinal cholesterol synthesis, including *Hmgcs1* and *Hmgcr* (Fig 4B and 4C). Next, we investigated whether there were any changes in the expression of genes regulating cholesterol esterification and basolateral transport. Expression of *Acat2* was increased only in the presence of both GSPE and CHY (Fig 4D), as was *Mttp* expression (Fig 4E). *Scarb1* expression was also increased by CHY and CHY+GSPE, compared to control (Fig 4F). Collectively, these results suggest that the CHY-treated animals are attempting to take in more cholesterol from both dietary and endogenous sources via increased *scarb1* expression, possibly as a compensatory mechanism consequential to significantly reduced luminal BA levels following CHY treatment. The results also indicate that there may be increased cholesterol synthesis within the enterocyte. Previous reports have suggested the presence of a substance within bile that normally inhibits intestinal steroidogenesis [50]. Therefore, a substantial decrease in BA uptake following CHY treatment would likely initiate increased cholesterol synthesis within the enterocyte. Newly synthesized cholesterol could then be esterified by Acat2 and subsequently loaded onto chylomicrons for export into the lymphatic system in the CHY+GSPE treated animals, which is consistent with previous reports showing that cholesterol synthesized in the gut enters the lymph, eventually becoming part of the circulating cholesterol pool [51]. Alternatively, CHY-treatment is known to cause intestinal cellular damage [52, 53] and since newly synthesized cholesterol is primarily used for structural purposes [50] it could be used to protect the intestine against CHY-induced damage and to help maintain intestinal cell membrane integrity.

**Fig 5:**
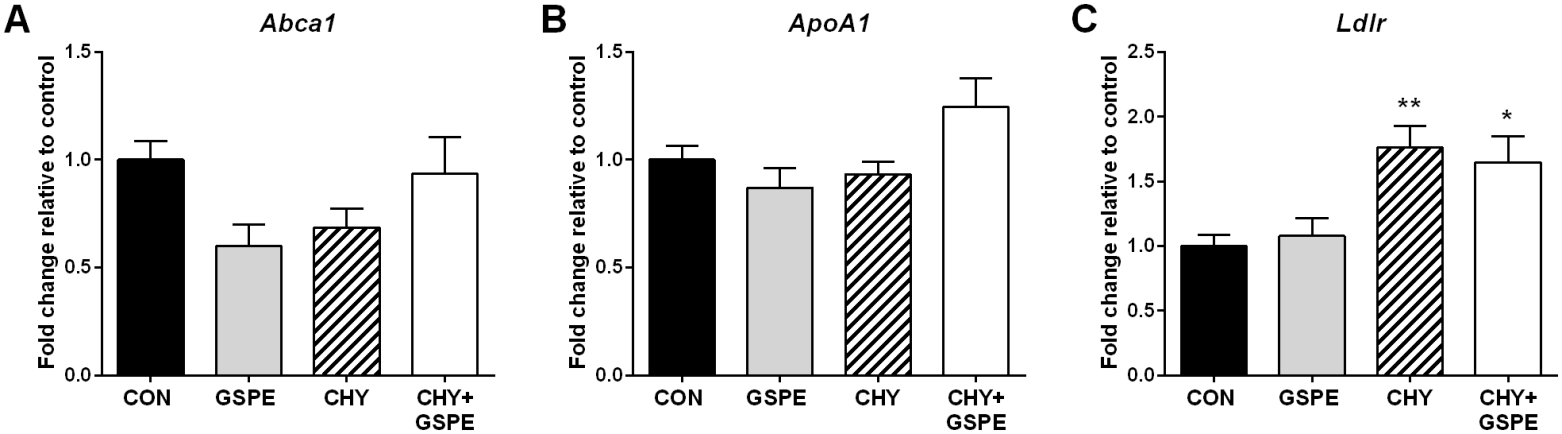
Expression of genes involved in basolateral intestinal cholesterol transport following treatments. Gene expression was analyzed for (A) *Abca1*, (B) *ApoA1*, and (C) *Ldlr*. Statistical differences are shown as: *p≤0.05, ** p≤0.01.

Basolateral cholesterol transporter gene expression was also evaluated in the intestine. The expression of *Abca1* and *ApoA1* remained unchanged following any of the treatments (Fig 5A and 5B), whereas *Ldlr* expression was increased following CHY and CHY+GSPE treatment (Fig 5C).

The small intestine is central to the regulation of whole-body cholesterol balance in mammals and is the second most active site for cholesterol synthesis [54–56]. Enterocytes are unusual in that they have three, rather than two, sources of cholesterol. Enterocytes uniquely absorb free cholesterol from the gut lumen, and they also share two ubiquitous sources with other cells, namely endogenous *de novo* synthesis and uptake of LDL-derived cholesterol from plasma. Increased cholesterol synthesis combined with increased uptake of LDL, via the Ldlr, in both the CHY-treated groups, could theoretically be a protective mechanism to maintain cholesterol homeostasis.

### GSPE counteracts the cholestyramine-induced increase in hepatic cholesterol and triglyceride synthesis

**Fig 6:**
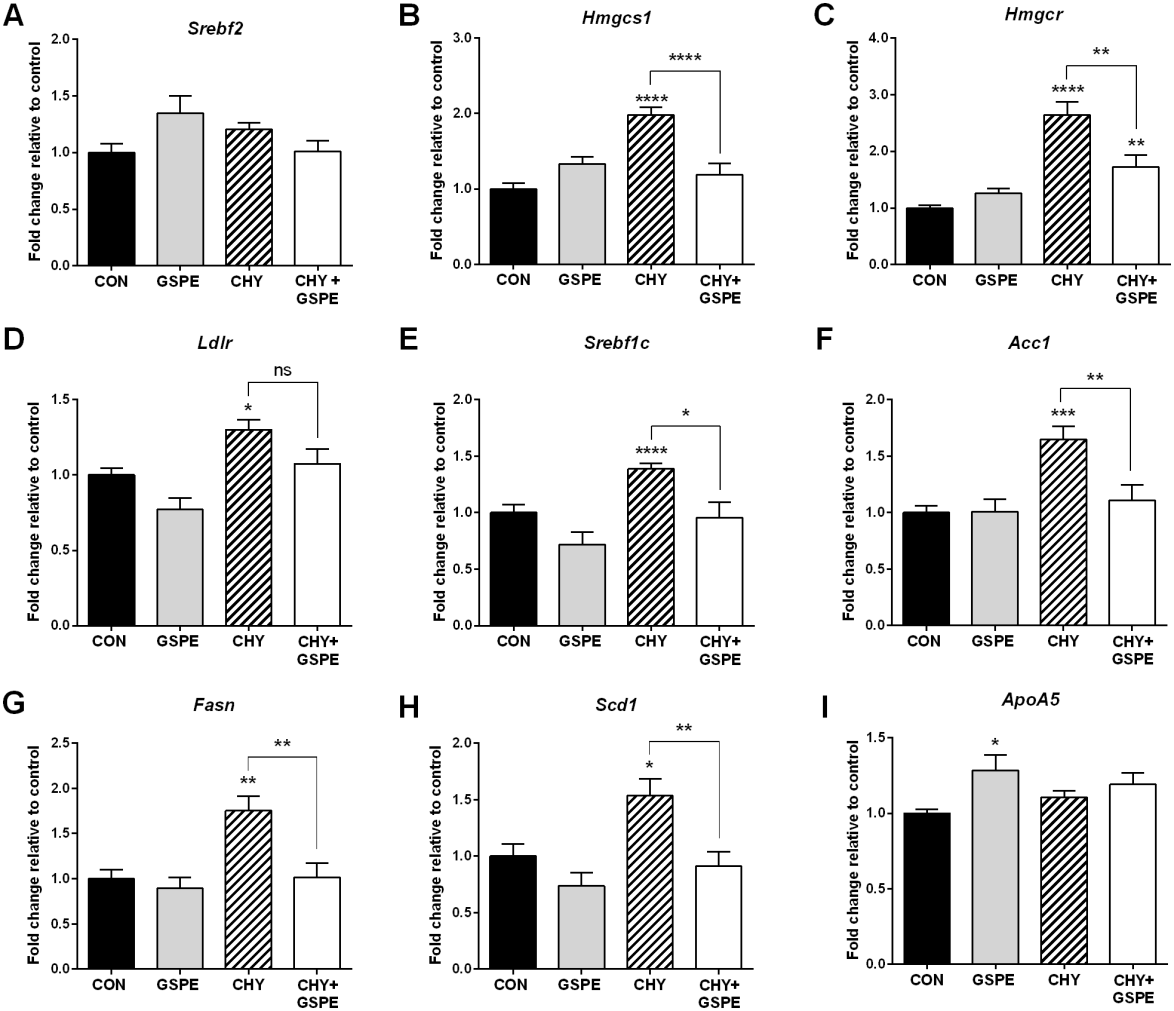
Hepatic cholesterol and lipogenic homeostatic gene expression following treatments. Gene expression was analyzed for (A) *Srebf2*, (B) *Hmgcs1*, (C) *Hmgcr*, (D) *Ldlr*, (E) *Srebf1c*, (F) *Acc1*, (G) *Fasn*, (H) *Scd1*, and (I) *ApoA5*. Statistical differences are shown as: *p≤0.05, ** p≤0.01, ***p≤0.001, **** p≤0.0001.

After confirming that CHY alters intestinal cholesterol synthesis, we next examined the effect on hepatic cholesterol synthesis. As shown in Fig 6A, the expression of *Srebf2* remained unchanged following any treatments, while *Hmgcs1* expression was significantly increased by CHY, but not GSPE treatment, compared to control (Fig 6A and 6B). Intriguingly, treatment with GSPE in combination with CHY attenuated the CHY-induced increase in *Hmgcs1* expression (Fig 6B). *Hmgcr* and *Ldlr* expression also followed a similar pattern with GSPE attenuating the CHY-induced increase (Fig 6C and 6D).

The expression of *Srebf1c* was significantly increased in the CHY-treated group, consistent with previous reports [48]. In this particular study, although trending downwards, GSPE did not significantly decrease *Srebf1c* expression (Fig 6E). Interestingly however, combined treatment with CHY+GSPE attenuated the CHY-induced increase returning levels back to control. To determine whether this impacted Srebp1c-target gene expression, we next examined the expression of *Fasn*, *Acc1*, and *Scd1*. In agreement with increased *Srebf1c* expression, their expression was also induced following CHY-treatment, which was again attenuated following CHY+GSPE treatment (Fig 6F, 6G and 6H). Additionally, expression of *ApoA5*, which has been shown to increase plasma TG clearance and decrease VLDL synthesis [57, 58] was increased by GSPE, but not by CHY or CHY+GSPE (Fig 6I).

The evidence presented herein collectively suggests that the combined treatment with CHY+GSPE may reverse the CHY-induced increase in hepatic cholesterol and triglyceride synthesis. The effects exerted by GSPE, may be initiated via the increased requirement for BA biosynthesis, consequential to decreased intestinal *Asbt* expression. This would be consistent with the fact that Asbt inhibition triggers a feed forward upregulation in hepatic BA synthesis [59, 60]. Additionally, the increase in *Srebf1c* expression following CHY treatment could be consequential to the lack of intestinally derived Fgf15. Under normal circumstances, Fgf15 represses Srebp1c and its downstream lipogenic target-gene transcription, including *Fasn* and *Acc1* (Reviewed in [32]). The absence of Fgf15 in the CHY-treated animals (Fig 1C) would therefore lead to a consequential increase in Srebp1c and lipogenesis, consistent with the observed increase in *Srebf1c*, *Acc1*, *Fasn* and *Scd-1* expression (Fig 6E, 6F, 6G and 6H). GSPE was previously shown to repress *Srebf1c* in an Fxr-Shp-dependent manner [39, 44]. Therefore, the GSPE-induced repression of *Srebf1c* could lead to decreased lipogenesis despite the absence of *Fgf15* in the CHY+GSPE-treated animals.

### GSPE and cholestyramine decrease serum bile acid, triglyceride and non-esterified fatty acid levels and increase fecal bile acid and lipid excretion

**Fig 7:**
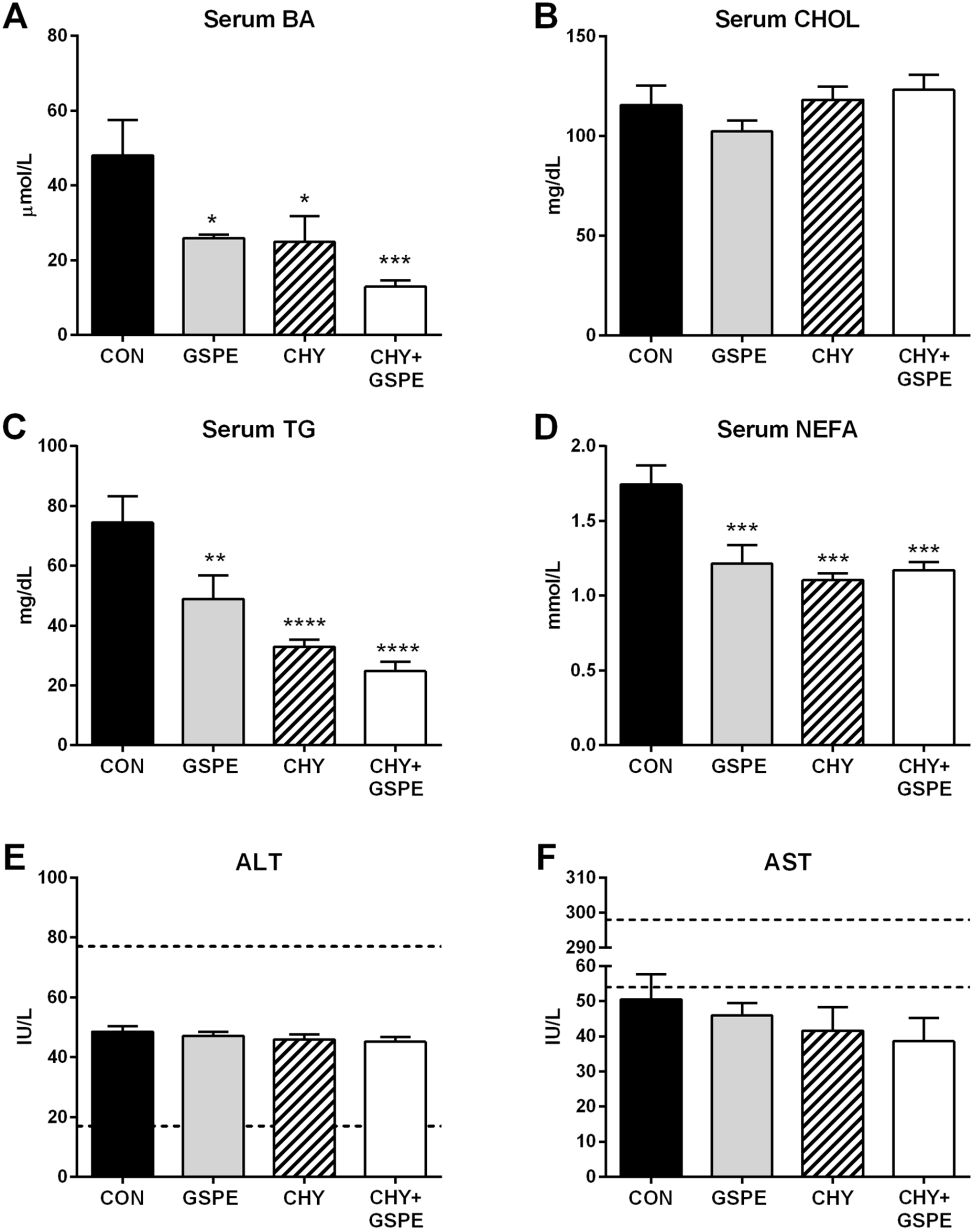
Serum Biochemical analysis following treatments. Serum analysis was performed for (A) bile acids (BA), (B) cholesterol (CHOL), (C) triglyceride (TG), (D) non-esterified fatty acids (NEFA), (E) alanine aminotransferase (ALT), and (F) aspartate aminotransferase (AST). Normal upper and lower limits for ALT and AST are represented by the dashed lines in (E) and (F). Statistical differences are shown as: *p≤0.05, **p≤0.01, ***p≤0.001, **** p≤0.0001.

Following gene expression analysis, we looked at the consequential physiological effects. Serum BA levels were significantly reduced following treatment with either GSPE or CHY (Fig 7A), and were further reduced in the CHY+GSPE group, compared to control. As shown in Fig 7B, no changes in serum cholesterol levels were observed by any treatment regime, which is in agreement with previous reports showing that serum cholesterol levels are unlikely to be altered by CHY in a normolipidemic state [48]. However, serum TG levels were significantly decreased by all treatments compared to control, with GSPE exerting a 34% decrease; CHY a 56% decrease; and CHY+GSPE a 66.7% decrease compared to control (Fig 7C). Combined treatment with CHY+GSPE exerted a 25% additional decrease in serum TG levels compared to CHY alone. Serum NEFAs were also decreased in all three treatment groups, compared to control (Fig 7D). No detrimental effects were exerted in the liver by any of the treatments, as evidenced by the fact that ALT and AST levels were unchanged (Fig 7E and 7F).

**Fig 8:**
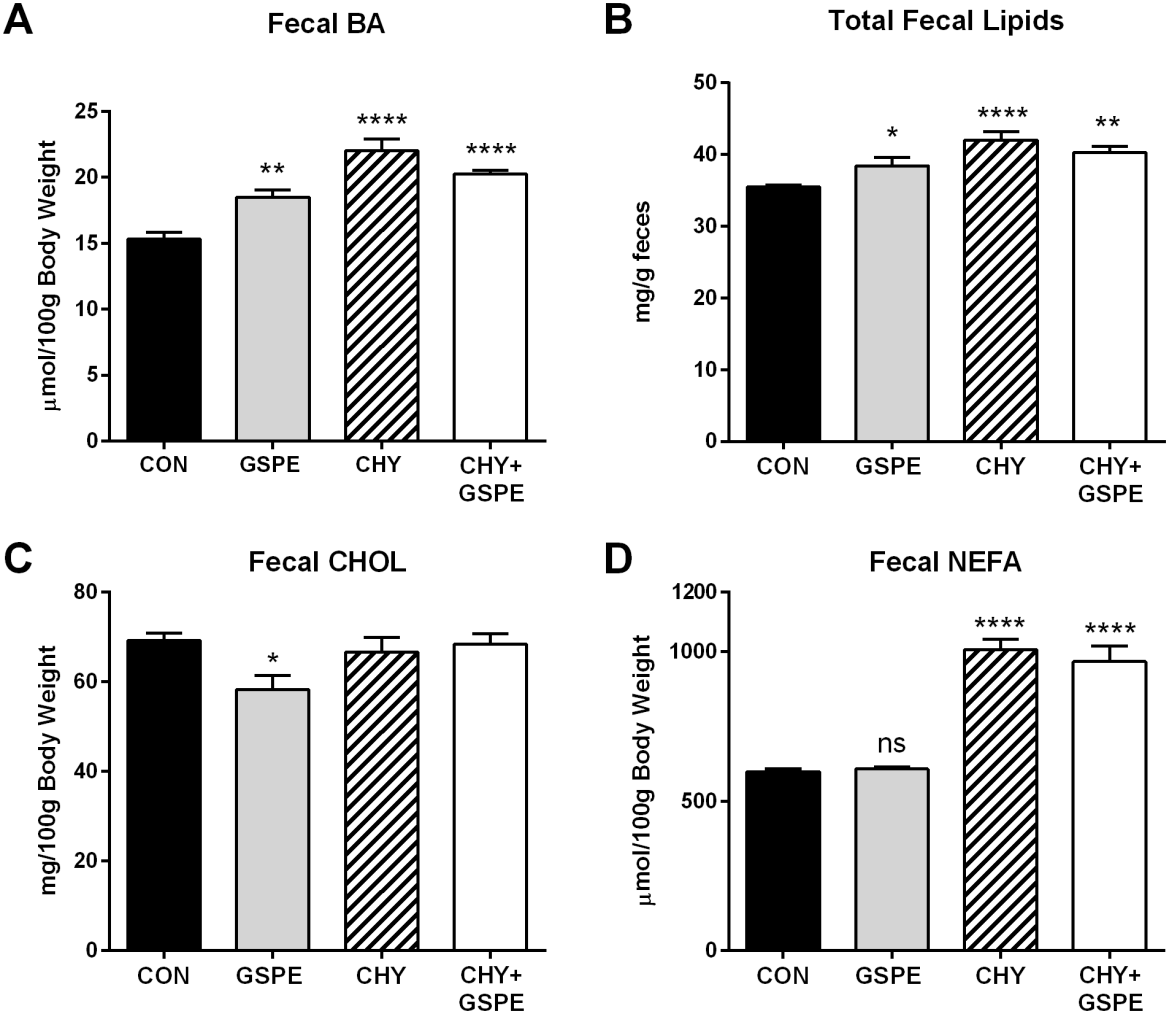
Fecal bile acid, cholesterol and lipid analysis following treatments. Feces were analyzed for (A) bile acids (BA), (B) total lipids, (C) cholesterol (CHOL), and (D) non-esterified fatty acids (NEFA). Statistical differences are shown as: *p≤0.05, **p≤0.01, **** p≤0.0001.

Finally, the effect on fecal BA and lipid excretion was determined. Fecal BA excretion was significantly increased by administration with GSPE, CHY and CHY+GSPE (Fig 8A) in agreement with the observed reduction in serum BA levels (Fig 7A). Total fecal lipids were also increased by all treatments compared to control (Fig 8B). Consistent with the observed decrease in *Abcg5* expression (Fig 3A), fecal cholesterol excretion was also decreased by GSPE (Fig 8C), possibly because cholesterol is being conserved to synthesize BAs. Fecal NEFA levels were also unchanged following GSPE administration in the control-fed animals, however, CHY treatment caused a significant increase in fecal NEFA excretion (Fig 8D), consistent with the reduced serum NEFA levels (Fig 7D). Colestilan, another BA sequestrant, also causes increased NEFA incorporation into biliary secretions subsequently increasing fecal excretion [61], therefore, CHY may cause the observed effect via a similar mechanism.

## Conclusion

Collectively, the data shows that GSPE and CHY, both independently and when combined differentially alter the expression of genes controlling BA, cholesterol and triglyceride homeostasis. Beginning in the intestine, GSPE decreases BA uptake by inhibiting *Asbt* expression, thereby limiting BA absorption via gene regulatory mechanisms. In contrast, CHY, by sequestering BAs, induces *Asbt* expression. Ultimately, both mechanisms of action lead to reduced serum BA levels and concomitant increased fecal BA excretion.

Importantly, CHY induces genes associated with cholesterol synthesis both in the intestine and liver. In comparison, GSPE co-administration selectively attenuates the CHY-induced increase in cholesterol synthetic gene expression in the liver, but not intestine. In addition, the findings suggest that cholesterol transport into the lumen of the intestine from the enterocyte, as well as from the lumen into the enterocyte is decreased by GSPE, but not CHY.

CHY and GSPE both increase BA biosynthesis independently, with a significant increase in *Cyp7a1* expression. In the presence of CHY+GSPE, *Cyp27a1* expression is significantly induced, as are *Cyp7a1* and *Cyp8b1*, which would theoretically lead to an increase in the production of both CA and CDCA. In the presence of GSPE only, neither *Cyp8b1* nor *Cyp27a1* were induced; while CHY increased *Cyp8b1* expression. Whether these gene changes lead to consequential changes in the BA pool composition requires further detailed analysis.

CHY increases hepatic lipogenic gene expression, whereas co-administration with GSPE attenuates these changes. Ultimately, both CHY and GSPE alone reduce serum TG levels, but they are reduced even further following co-administration, which is probably due to the combination of increased BA biosynthesis and decreased lipogenesis induced by GSPE. The findings from this study suggest that the decrease in *Fgf15* expression induced by treatment with CHY, GSPE or CHY+GSPE, lead to differential hepatic gene regulatory effects. In the CHY-treated animals, the absence of *Fgf15* facilitates relief from repression on Srebp1c, leading to not only increased *Srebf1c* gene expression, but also its downstream targets, including *Acc1*, *Fasn* and *Scd-1*. In contrast, due to the direct hepatic repression of *Srebf1c* by GSPE, the CHY-induced increase in lipogenic gene expression is attenuated, despite the lack of intestinally-derived *Fgf15* in the animals treated with both CHY+GSPE. Previous studies showed that the liver is the main site for protective effects of BA-Fxr signaling to combat lipid accumulation [62]. Notably, organ-specific deletion of hepatic Fxr resulted in lipid accumulation, which occurred independently from Fxr-Fgf15 signaling in the intestine [62]. These observations are consistent with the results from this study in the CHY+GSPE treated animals showing that although intestinal *Fgf15* expression is significantly reduced, GSPE still exerts a repressive effect on hepatic *Srebf1c* expression, an effect which we previously showed was mechanistically mediated in an Fxr-Shp dependent manner [39, 44].

Although we did not evaluate the protein expression levels of these gene products in this study, it is possible not only to conclude that intraluminal availability of BAs alters the expression of various genes relating to BA and lipid metabolism in the presence of CHY, but also that GSPE exerts independent effects to modulate BA and lipid metabolism resulting in physiological effects such as reduced lipogenesis and consequently further decreasing serum TG levels.

Due to the fact that BA sequestrants are not metabolized, there are no reported drug-drug interactions [22]. However, BA sequestrants are positively charged and they may bind non-specifically to co-administered drugs, particularly those that are acidic, ultimately reducing their bioavailability [22]. The differential effects exerted by GSPE both in the intestine and liver in the presence of CHY clearly indicate that CHY does not interfere with the absorption of GSPE nor its subsequent molecular actions.

Although BA sequestrant monotherapy effectively lowers LDL-cholesterol, combined therapy, e.g. with statins, is common due to their complementary mechanisms of action for those patients who require more aggressive lipid-lowering therapy. BA depletion leads to increased HMG CoA-reductase activity, therefore, interfering with this enzyme results in additive and complementary effects on the lipid profile. Results presented herein show that GSPE exerts beneficial effects by decreasing HMG CoA-reductase gene expression, selectively in the liver, when combined with CHY. Therefore combination therapy with CHY and GSPE may prove particularly efficacious and beneficial in the amelioration of CVD, clearly warranting further investigation. Although statins are the first-line drug to treat hypercholesterolemia, some patients are statin intolerant, for example those suffering from rhabdomyolysis or certain liver diseases, and they may benefit from combined therapy with CHY and GSPE. Furthermore, patients with hypertriglyceridemia who would not otherwise be prescribed CHY may benefit from the addition of GSPE with CHY since GSPE exerts a hypotriglyceridemic effect. Clearly further studies are needed to determine whether CHY in combination with GSPE as a lipid-lowering therapy can improve cardiovascular outcomes, slow atherosclerotic progression or reduce plaque build-up.

In conclusion, this study provides novel and innovative insight into the molecular regulatory interactions between GSPE and CHY. A natural product, such as GSPE, that can induce *Cyp7a1* gene expression, inhibit uptake of BAs produced in the process, and reduce lipogenesis may be valuable as a potential combinational therapy with CHY for the treatment of dyslipidemia.

## Supporting Information

S1 Table qPCR Primer and Probe Sequences. (XLSX)

## Abbreviations

BA: Bile acid
CHY: Cholestyramine
GSPE: Grape seed procyanidin extract
TG: Triglyceride

## References

[1] Centers for Disease Control and Prevention National Center for Health Statistics. Underlying cause of death 1999–2014 CDC WONDER Online database: Vital Statistics Cooperative Program; 2015. Available: http://wonder.cdc.gov/ucd-icd10.html.

[2] Makishima M, Okamoto AY, Repa JJ, Tu H, Learned RM, Luk A, et al Identification of a nuclear receptor for bile acids. Science. 1999;284(5418):1362–5. Epub 1999/05/21. . 10334992 10.1126/science.284.5418.1362

[3] Goodwin B, Jones SA, Price RR, Watson MA, McKee DD, Moore LB, et al A regulatory cascade of the nuclear receptors FXR, SHP-1, and LRH-1 represses bile acid biosynthesis. Molecular cell. 2000;6(3):517–26. Epub 2000/10/13. . 11030332 10.1016/s1097-2765(00)00051-4

[4] Watanabe M, Houten SM, Wang L, Moschetta A, Mangelsdorf DJ, Heyman RA, et al Bile acids lower triglyceride levels via a pathway involving FXR, SHP, and SREBP-1c. The Journal of clinical investigation. 2004;113(10):1408–18. Epub 2004/05/18. 10.1172/JCI21025 15146238 PMC406532

[5] Dawson PA, Haywood J, Craddock AL, Wilson M, Tietjen M, Kluckman K, et al Targeted deletion of the ileal bile acid transporter eliminates enterohepatic cycling of bile acids in mice. The Journal of biological chemistry. 2003;278(36):33920–7. Epub 2003/06/24. 10.1074/jbc.M306370200 . 12819193

[6] Dawson PA, Lan T, Rao A. Bile acid transporters. Journal of lipid research. 2009;50(12):2340–57. Epub 2009/06/06. 10.1194/jlr.R900012-JLR200 19498215 PMC2781307

[7] Li H, Chen F, Shang Q, Pan L, Shneider BL, Chiang JY, et al FXR-activating ligands inhibit rabbit ASBT expression via FXR-SHP-FTF cascade. American journal of physiology Gastrointestinal and liver physiology. 2005;288(1):G60–6. Epub 2004/12/14. 10.1152/ajpgi.00170.2004 . 15591588

[8] Praslickova D, Torchia EC, Sugiyama MG, Magrane EJ, Zwicker BL, Kolodzieyski L, et al The ileal lipid binding protein is required for efficient absorption and transport of bile acids in the distal portion of the murine small intestine. PloS one. 2012;7(12):e50810 Epub 2012/12/20. 10.1371/journal.pone.0050810 23251388 PMC3519535

[9] Dawson PA, Hubbert M, Haywood J, Craddock AL, Zerangue N, Christian WV, et al The heteromeric organic solute transporter alpha-beta, Ostalpha-Ostbeta, is an ileal basolateral bile acid transporter. The Journal of biological chemistry. 2005;280(8):6960–8. Epub 2004/11/26. 10.1074/jbc.M412752200 15563450 PMC1224727

[10] Ishibashi S, Schwarz M, Frykman PK, Herz J, Russell DW. Disruption of cholesterol 7alpha-hydroxylase gene in mice. I. Postnatal lethality reversed by bile acid and vitamin supplementation. The Journal of biological chemistry. 1996;271(30):18017–23. Epub 1996/07/26. . 8663429 10.1074/jbc.271.30.18017

[11] Inagaki T, Choi M, Moschetta A, Peng L, Cummins CL, McDonald JG, et al Fibroblast growth factor 15 functions as an enterohepatic signal to regulate bile acid homeostasis. Cell metabolism. 2005;2(4):217–25. Epub 2005/10/11. 10.1016/j.cmet.2005.09.001 . 16213224

[12] Yu C, Wang F, Jin C, Huang X, McKeehan WL. Independent repression of bile acid synthesis and activation of c-Jun N-terminal kinase (JNK) by activated hepatocyte fibroblast growth factor receptor 4 (FGFR4) and bile acids. The Journal of biological chemistry. 2005;280(18):17707–14. Epub 2005/03/08. 10.1074/jbc.M411771200 . 15750181

[13] Holt JA, Luo G, Billin AN, Bisi J, McNeill YY, Kozarsky KF, et al Definition of a novel growth factor-dependent signal cascade for the suppression of bile acid biosynthesis. Genes & development. 2003;17(13):1581–91. Epub 2003/06/20. 10.1101/gad.1083503 12815072 PMC196131

[14] Bernhardsson C, Bjorkhem I, Danielsson H, Wikvall K. 12alpha-hydroxylation of 7alpha-hydroxy-4-cholesten-3-one by a reconstituted system from rat liver microsomes. Biochemical and biophysical research communications. 1973;54(3):1030–8. Epub 1973/10/01. . 4148126 10.1016/0006-291x(73)90797-3

[15] Eggertsen G, Olin M, Andersson U, Ishida H, Kubota S, Hellman U, et al Molecular cloning and expression of rabbit sterol 12alpha-hydroxylase. The Journal of biological chemistry. 1996;271(50):32269–75. Epub 1996/12/13. . 8943286 10.1074/jbc.271.50.32269

[16] Pandak WM, Bohdan P, Franklund C, Mallonee DH, Eggertsen G, Bjorkhem I, et al Expression of sterol 12alpha-hydroxylase alters bile acid pool composition in primary rat hepatocytes and in vivo. Gastroenterology. 2001;120(7):1801–9. Epub 2001/05/29. . 11375960 10.1053/gast.2001.24833

[17] Li-Hawkins J, Gafvels M, Olin M, Lund EG, Andersson U, Schuster G, et al Cholic acid mediates negative feedback regulation of bile acid synthesis in mice. The Journal of clinical investigation. 2002;110(8):1191–200. Epub 2002/10/24. 10.1172/JCI16309 12393855 PMC150802

[18] Thomas C, Pellicciari R, Pruzanski M, Auwerx J, Schoonjans K. Targeting bile-acid signalling for metabolic diseases. Nature reviews Drug discovery. 2008;7(8):678–93. Epub 2008/08/02. 10.1038/nrd2619 . 18670431

[19] Chiang JY. Regulation of bile acid synthesis. Frontiers in bioscience: a journal and virtual library. 1998;3:d176–93. Epub 1998/02/27. . 9450986 10.2741/a273

[20] Schwarz M, Lund EG, Lathe R, Bjorkhem I, Russell DW. Identification and characterization of a mouse oxysterol 7alpha-hydroxylase cDNA. The Journal of biological chemistry. 1997;272(38):23995–4001. Epub 1997/09/20. . 9295351 10.1074/jbc.272.38.23995

[21] Shepherd J. Mechanism of action of bile acid sequestrants and other lipid-lowering drugs. Cardiology. 1989;76 Suppl 1:65–71; discussion -4. Epub 1989/01/01. . 2713876 10.1159/000174548

[22] Insull W Jr. Clinical utility of bile acid sequestrants in the treatment of dyslipidemia: a scientific review. Southern medical journal. 2006;99(3):257–73. Epub 2006/03/24. 10.1097/01.smj.0000208120.73327.db . 16553100

[23] Dietschy JM, Weis HJ. Cholesterol synthesis by the gastrointestinal tract. The American journal of clinical nutrition. 1971;24(1):70–6. Epub 1971/01/01. . 4992518 10.1093/ajcn/24.1.70

[24] Dietschy JM, Gamel WG. Cholesterol synthesis in the intestine of man: regional differences and control mechanisms. The Journal of clinical investigation. 1971;50(4):872–80. Epub 1971/04/01. 10.1172/JCI106559 4993859 PMC292002

[25] Hua X, Yokoyama C, Wu J, Briggs MR, Brown MS, Goldstein JL, et al SREBP-2, a second basic-helix-loop-helix-leucine zipper protein that stimulates transcription by binding to a sterol regulatory element. Proceedings of the National Academy of Sciences of the United States of America. 1993;90(24):11603–7. Epub 1993/12/15. 7903453 10.1073/pnas.90.24.11603 PMC48032

[26] Cases S, Novak S, Zheng YW, Myers HM, Lear SR, Sande E, et al ACAT-2, a second mammalian acyl-CoA:cholesterol acyltransferase. Its cloning, expression, and characterization. The Journal of biological chemistry. 1998;273(41):26755–64. Epub 1998/10/03. . 9756919 10.1074/jbc.273.41.26755

[27] Wetterau JR, Aggerbeck LP, Laplaud PM, McLean LR. Structural properties of the microsomal triglyceride-transfer protein complex. Biochemistry. 1991;30(18):4406–12. Epub 1991/05/07. . 2021632 10.1021/bi00232a006

[28] Wetterau JR, Combs KA, Spinner SN, Joiner BJ. Protein disulfide isomerase is a component of the microsomal triglyceride transfer protein complex. The Journal of biological chemistry. 1990;265(17):9800–7. Epub 1990/06/15. . 2351674

[29] Wetterau JR, Zilversmit DB. Purification and characterization of microsomal triglyceride and cholesteryl ester transfer protein from bovine liver microsomes. Chemistry and physics of lipids. 1985;38(1–2):205–22. Epub 1985/08/30. . 4064222 10.1016/0009-3084(85)90068-4

[30] Hayashi AA, Webb J, Choi J, Baker C, Lino M, Trigatti B, et al Intestinal SR-BI is upregulated in insulin-resistant states and is associated with overproduction of intestinal apoB48-containing lipoproteins. American journal of physiology Gastrointestinal and liver physiology. 2011;301(2):G326–37. Epub 2011/05/07. 10.1152/ajpgi.00425.2010 . 21546579

[31] Brunham LR, Kruit JK, Iqbal J, Fievet C, Timmins JM, Pape TD, et al Intestinal ABCA1 directly contributes to HDL biogenesis in vivo. The Journal of clinical investigation. 2006;116(4):1052–62. Epub 2006/03/18. 10.1172/JCI27352 16543947 PMC1401485

[32] Jahn D, Rau M, Hermanns HM, Geier A. Mechanisms of enterohepatic fibroblast growth factor 15/19 signaling in health and disease. Cytokine & growth factor reviews. 2015;26(6):625–35. Epub 2015/08/08. 10.1016/j.cytogfr.2015.07.016 . 26250749

[33] Bhatnagar S, Damron HA, Hillgartner FB. Fibroblast growth factor-19, a novel factor that inhibits hepatic fatty acid synthesis. The Journal of biological chemistry. 2009;284(15):10023–33. Epub 2009/02/24. 10.1074/jbc.M808818200 19233843 PMC2665057

[34] Seok S, Kanamaluru D, Xiao Z, Ryerson D, Choi SE, Suino-Powell K, et al Bile acid signal-induced phosphorylation of small heterodimer partner by protein kinase Czeta is critical for epigenomic regulation of liver metabolic genes. The Journal of biological chemistry. 2013;288(32):23252–63. Epub 2013/07/05. 10.1074/jbc.M113.452037 23824184 PMC3743497

[35] Kobayashi M, Ikegami H, Fujisawa T, Nojima K, Kawabata Y, Noso S, et al Prevention and treatment of obesity, insulin resistance, and diabetes by bile acid-binding resin. Diabetes. 2007;56(1):239–47. Epub 2006/12/29. 10.2337/db06-0353 . 17192488

[36] Out C, Groen AK, Brufau G. Bile acid sequestrants: more than simple resins. Current opinion in lipidology. 2012;23(1):43–55. Epub 2011/12/22. 10.1097/MOL.0b013e32834f0ef3 . 22186660

[37] Hou R, Goldberg AC. Lowering low-density lipoprotein cholesterol: statins, ezetimibe, bile acid sequestrants, and combinations: comparative efficacy and safety. Endocrinology and metabolism clinics of North America. 2009;38(1):79–97. Epub 2009/02/17. 10.1016/j.ecl.2008.11.007 . 19217513

[38] Crouse JR 3rd. Hypertriglyceridemia: a contraindication to the use of bile acid binding resins. The American journal of medicine. 1987;83(2):243–8. Epub 1987/08/01. . 3618626 10.1016/0002-9343(87)90692-9

[39] Del Bas JM, Ricketts ML, Vaque M, Sala E, Quesada H, Ardevol A, et al Dietary procyanidins enhance transcriptional activity of bile acid-activated FXR in vitro and reduce triglyceridemia in vivo in a FXR-dependent manner. Molecular nutrition & food research. 2009;53(7):805–14. Epub 2009/06/06. 10.1002/mnfr.200800364 . 19496086 PMC4142053

[40] Heidker RM, Caiozzi GC, Ricketts ML. Dietary procyanidins selectively modulate intestinal farnesoid X receptor-regulated gene expression to alter enterohepatic bile acid recirculation: elucidation of a novel mechanism to reduce triglyceridemia. Molecular nutrition & food research. 2016;60(4):727–36. Epub 2016/01/01. 10.1002/mnfr.201500795 . 26718753

[41] Downing LE, Heidker RM, Caiozzi GC, Wong BS, Rodriguez K, Del Rey F, et al A Grape Seed Procyanidin Extract Ameliorates Fructose-Induced Hypertriglyceridemia in Rats via Enhanced Fecal Bile Acid and Cholesterol Excretion and Inhibition of Hepatic Lipogenesis. PloS one. 2015;10(10):e0140267 Epub 2015/10/13. 10.1371/journal.pone.0140267 26458107 PMC4601771

[42] Clarke TC, Black LI, Stussman BJ, Barnes PM, Nahin RL. Trends in the use of complementary health approaches among adults: United States, 2002–2012. National health statistics reports. 2015;(79):1–16. Epub 2015/02/12. 25671660 PMC4573565

[43] Yamakoshi J, Saito M, Kataoka S, Kikuchi M. Safety evaluation of proanthocyanidin-rich extract from grape seeds. Food and chemical toxicology: an international journal published for the British Industrial Biological Research Association. 2002;40(5):599–607. Epub 2002/04/17. . 11955665 10.1016/s0278-6915(02)00006-6

[44] Del Bas JM, Ricketts ML, Baiges I, Quesada H, Ardevol A, Salvado MJ, et al Dietary procyanidins lower triglyceride levels signaling through the nuclear receptor small heterodimer partner. Molecular nutrition & food research. 2008;52(10):1172–81. Epub 2008/08/23. 10.1002/mnfr.200800054 . 18720348

[45] Modica S, Murzilli S. and Moschetta A. Characterizing Bile Acid and Lipid Metabolism in the Liver and Gastrointestinal Tract of Mice. Current Protocols in Mouse Biology. 2011;1:289–321. 10.1002/9780470942390.mo100226 26069056

[46] Lundasen T, Galman C, Angelin B, Rudling M. Circulating intestinal fibroblast growth factor 19 has a pronounced diurnal variation and modulates hepatic bile acid synthesis in man. Journal of internal medicine. 2006;260(6):530–6. Epub 2006/11/23. 10.1111/j.1365-2796.2006.01731.x . 17116003

[47] Vlahcevic ZR, Eggertsen G, Bjorkhem I, Hylemon PB, Redford K, Pandak WM. Regulation of sterol 12alpha-hydroxylase and cholic acid biosynthesis in the rat. Gastroenterology. 2000;118(3):599–607. Epub 2000/03/04. . 10702212 10.1016/s0016-5085(00)70267-8

[48] Kamisako T, Ogawa H, Yamamoto K. Effect of cholesterol, cholic acid and cholestyramine administration on the intestinal mRNA expressions related to cholesterol and bile acid metabolism in the rat. Journal of gastroenterology and hepatology. 2007;22(11):1832–7. Epub 2007/05/15. 10.1111/j.1440-1746.2007.04910.x . 17498222

[49] Feng D, Ohlsson L, Duan RD. Curcumin inhibits cholesterol uptake in Caco-2 cells by down-regulation of NPC1L1 expression. Lipids in health and disease. 2010;9:40 Epub 2010/04/21. 10.1186/1476-511X-9-40 20403165 PMC2865464

[50] Dietschy JM, Siperstein MD. Cholesterol synthesis by the gastrointestinal tract: localization and mechanisms of control. The Journal of clinical investigation. 1965;44(8):1311–27. Epub 1965/08/01. 10.1172/JCI105237 5835437 PMC292609

[51] Lindsey CA Jr., Wilson JD. Evidence for a Contribution by the Intestinal Wall to the Serum Cholesterol of the Rat. Journal of lipid research. 1965;6:173–81. Epub 1965/04/01. . 14328424

[52] Cassidy MM, Lightfoot FG, Grau LE, Roy T, Story JA, Kritchevsky D, et al Effect of bile salt-binding resins on the morphology of rat jejunum and colon. A scanning electron microscopy study. Digestive diseases and sciences. 1980;25(7):504–12. Epub 1980/07/01. . 7389538 10.1007/BF01315212

[53] Cassidy MM, Lightfoot FG, Grau L, Satchitanandum S, Vahouny GV. Lipid accumulation in jejunal and colonic mucosa following chronic cholestyramine (Questran) feeding. Digestive diseases and sciences. 1985;30(5):468–76. Epub 1985/05/01. . 3987478 10.1007/BF01318181

[54] Dietschy JM. The role of bile salts in controlling the rate of intestinal cholesterogenesis. The Journal of clinical investigation. 1968;47(2):286–300. Epub 1968/02/01. 10.1172/JCI105725 4966200 PMC297171

[55] Dietschy JM, Wilson JD. Cholesterol synthesis in the squirrel monkey: relative rates of synthesis in various tissues and mechanisms of control. The Journal of clinical investigation. 1968;47(1):166–74. Epub 1968/01/01. 10.1172/JCI105706 16695938 PMC297157

[56] Srere P, Chaikoff I, Treitman S, Burstein L. The extrahepatic synthesis of cholesterol. Journal of Biological Chemistry. 1950;182(2):629–34.

[57] O'Brien PJ, Alborn WE, Sloan JH, Ulmer M, Boodhoo A, Knierman MD, et al The novel apolipoprotein A5 is present in human serum, is associated with VLDL, HDL, and chylomicrons, and circulates at very low concentrations compared with other apolipoproteins. Clinical chemistry. 2005;51(2):351–9. Epub 2004/11/06. 10.1373/clinchem.2004.040824 . 15528295

[58] van der Vliet HN, Schaap FG, Levels JH, Ottenhoff R, Looije N, Wesseling JG, et al Adenoviral overexpression of apolipoprotein A-V reduces serum levels of triglycerides and cholesterol in mice. Biochemical and biophysical research communications. 2002;295(5):1156–9. Epub 2002/07/24. . 12135615 10.1016/s0006-291x(02)00808-2

[59] Li H, Xu G, Shang Q, Pan L, Shefer S, Batta AK, et al Inhibition of ileal bile acid transport lowers plasma cholesterol levels by inactivating hepatic farnesoid X receptor and stimulating cholesterol 7 alpha-hydroxylase. Metabolism: clinical and experimental. 2004;53(7):927–32. Epub 2004/07/16. . 15254889 10.1016/j.metabol.2004.01.017

[60] West KL, Zern TL, Butteiger DN, Keller BT, Fernandez ML. SC-435, an ileal apical sodium co-dependent bile acid transporter (ASBT) inhibitor lowers plasma cholesterol and reduces atherosclerosis in guinea pigs. Atherosclerosis. 2003;171(2):201–10. Epub 2003/12/04. . 14644388 10.1016/j.atherosclerosis.2003.08.019

[61] Sugimoto-Kawabata K, Shimada H, Sakai K, Suzuki K, Kelder T, Pieterman EJ, et al Colestilan decreases weight gain by enhanced NEFA incorporation in biliary lipids and fecal lipid excretion. Journal of lipid research. 2013;54(5):1255–64. Epub 2013/02/26. 10.1194/jlr.M032839 23434610 PMC3622322

[62] Schmitt J, Kong B, Stieger B, Tschopp O, Schultze SM, Rau M, et al Protective effects of farnesoid X receptor (FXR) on hepatic lipid accumulation are mediated by hepatic FXR and independent of intestinal FGF15 signal. Liver international: official journal of the International Association for the Study of the Liver. 2015;35(4):1133–44. Epub 2014/08/27. 10.1111/liv.12456 25156247 PMC4146754

